# *Andrographis paniculata* Dosage Forms and Advances in Nanoparticulate Delivery Systems: An Overview

**DOI:** 10.3390/molecules27196164

**Published:** 2022-09-20

**Authors:** Subashini Raman, Vikneswaran Murugaiyah, Thaigarajan Parumasivam

**Affiliations:** 1 School of Pharmaceutical Sciences, Universiti Sains Malaysia, Penang 11800, Malaysia; 2 Centre for Drug Research, Universiti Sains Malaysia, Penang 11800, Malaysia

**Keywords:** *Andrographis paniculata*, dosage form, solid, liquid, gaseous, nanoparticles

## Abstract

*Andrographis paniculata* is a well-known Asian medicinal plant with a major phytoconstituent of diterpene lactones, such as andrographolide, 14-deoxyandrographolide, and neoandrographolide. A World Health Organization (WHO) monograph on selected medicinal plants showed that *A. paniculata* extracts and its major diterpene lactones have promising anti-inflammatory, antidiabetic, antimalarial, anticancer, antifungal, antibacterial, antioxidant, and hypoglycemic activities. However, these active phytochemicals have poor water solubility and bioavailability when delivered in a conventional dosage form. These biological barriers can be mitigated if the extract or isolated compound are delivered as nanoparticles. This review discusses existing studies and marketed products of *A. paniculata* in solid, liquid, semi-solid, and gaseous dosage forms, either as an extract or isolated pure compounds, as well as their deficits in reaching maximum bioavailability. The pharmaceutics and pharmacological activity of *A. paniculata* as a nano-delivery system are also discussed.

## 1. Introduction

In the era of modern pharmacy, natural products or medicinal plants have gained much attention among researchers, due to their affordability and the acceptability of plant bioactive compounds for the treatment of modern or manmade diseases, with lower side effects. The *Andrographis paniculata (Burm.f.) Nees* is an herbaceous plant from the Acanthaceae family [1,2]. *A. paniculata* is also known as “Kalmegh” in hindi or “Hempedu bumi” in Malay. It is native to Taiwan, Mainland China, and India [2], and widely grown in Southeast Asian countries, including Malaysia. It has a strong background history of therapeutic usage in traditional Indian and Ayurvedic medicine, as well as traditional Chinese medicines [3,4,5].

In Ayurveda, *A. paniculata* is commonly used to treat sore throat, flu, liver disorders, jaundice, antispasmodic, malaria, eczema, intestinal worm infestation, and gonorrhoea [3,6]. On the other hand, the plant is used to remove body heat such as fevers and toxins, besides treating diarrhea, laryngitis, gastric infections, and inflammation in Chinese Medicine [7,8]. In contemporary pharmacology, *A. paniculata* acts as an immune system stimulant, treats pharyngotonsillitis, myocardial ischemia, and is anticancer, antimicrobial, anti-inflammatory, anti-HIV, and many more [1,9]. Recent studies have also discovered that *A. paniculata* can be used to treat SARS-CoV-2 [10].

### 1.1. Botanical Description of A. paniculata

**Figure 1:**
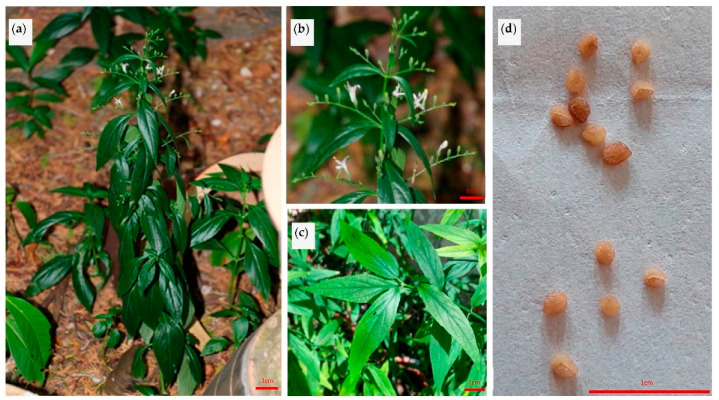
Images of *Andrographis paniculata*: (**a**) whole plants, (**b**) flowers, (**c**) leaves, and (**d**) seed. Bar = 1 cm.

*A. paniculata* (Figure 1), also known as king of bitters, is native to Taiwan, mainland China, and India. It is also widely distributed in many tropical and subtropical countries including Malaysia, Indonesia, Sri Lanka, Thailand, and Vietnam [1,2]. The plant height is about 30–110 cm with a dark green stem, glabrous leaves, and white with rose-purple spots on the petals. Its flowering and fruiting period is between December and April [2].

### 1.2. Major Phytochemicals

**Figure 2:**
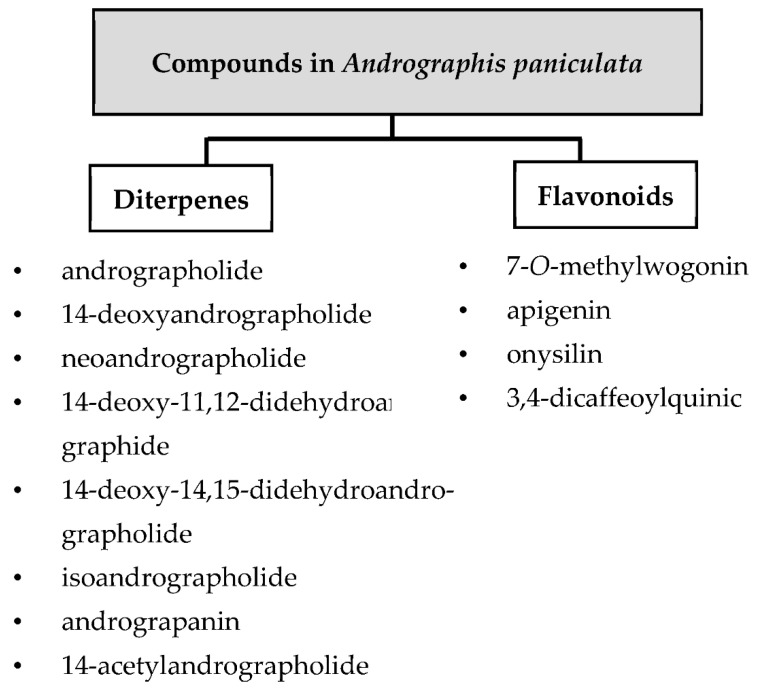
List of compounds isolated from *A. paniculata* [13,15,16,17,18,19].

**Figure 3:**
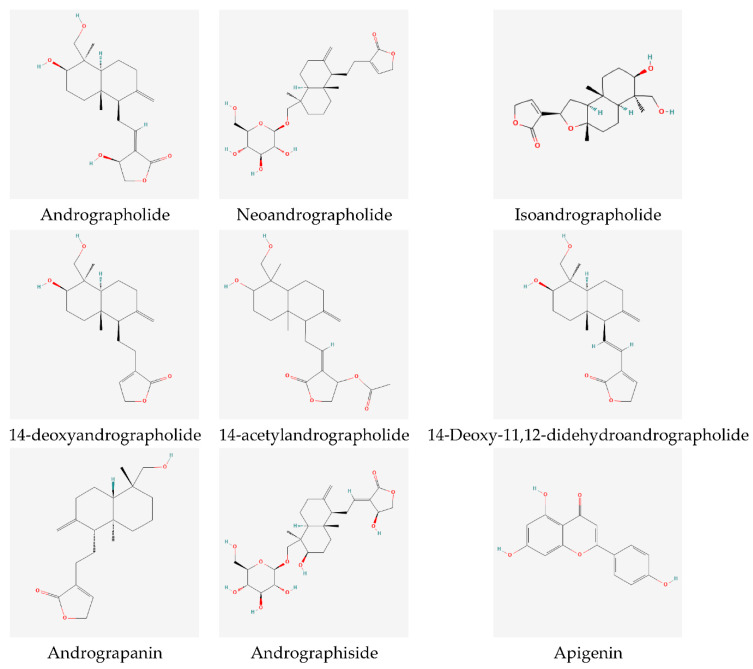
Chemical structures of active components in *A. paniculata* [16,18,20].

Scientists have isolated and characterized various bioactive compounds from the plant and have also extensively studied their therapeutic efficacy and pharmacological activities [3,11,12]. Over 20 diterpenes, 10 flavonoids, xanthones, noriridoides, and other compounds have been isolated from ethanol and methanol extracts [13,14,15]. There are nine major diterpenes and four typical flavonoids, as shown in Figure 2 and Figure 3. The most prominent compound with the highest quantity from the leaves is andrographolide (diterpenoids). This compound has also been extracted from the aerial parts or whole plant and is easily isolated from the crude extracts into a solid crystal [13,14]. Various dosage forms have been explored for the delivery of andrographolide, either as an extract or as a pure compound.

This review focuses on the current advances in the formulation approaches of *A. paniculata* in solid, liquid, semi-solid, and gaseous dosage forms. The pharmacological aspects of these formulations are also discussed, followed by advances in nanotechnology approaches for an enhanced therapeutic effect of andrographolide.

## 2. Conventional Dosage Forms

Each and every active pharmaceutical ingredient or medicinal plant extract compound can be formulated into various specialized dosage forms for patient consumption. Dosage forms can be classified into solid, semi-solid, liquid, and gas dosage forms [21]. In order to identify the presence of different dosage forms in *A. paniculata*, a literature search was performed using the keywords *Andrographis paniculata* and Kalmegh. The search resulted in approximately 5000 articles from both Scopus (2623) and Elsevier (2382), and the results were narrowed down to conventional dosage forms. In reference to the search, we encountered a wide range of solid dosage forms, such as tablets and capsules of andrographolide and *A. paniculata* extract. However, there are a few studies on liquid dosage forms and marketed products using andrographolide and *A. paniculata* extract. The same goes for semi-solid dosage forms such as patches, creams, or gels; meanwhile, there are almost no reports on gaseous dosage forms, such as aerosols.

### 2.1. Solid Dosage Forms: Tablets, Capsules, and Pills

In the pharmaceutical industry, solid dosage forms are most commonly prescribed and preferred by most medical practitioners, due to their accurate dosing, superior stability, and high precision [22,23]. For instance, the Therapeutic Goods Administration (TGA), Australia approved 59 solid dosage form products in October 2015 containing *A. paniculata* [24]. The listed products were mainly film-coated tablets, sugar-coated tablets, hard and soft capsules, enteric-coated capsules, and granules. Solid dosage forms, either as a mono or combination therapy, are also available in China and India [25,26].

In China, *A. paniculata* extract tablets are marketed as Chuan Xin Lian, Xiaoyan Lidan and Kan Jang tablets [27,28]. While in India, it can be found under names such as Green Milk Concepts Kalmegh tablets, Organic India Kalmegh 60 N Veg Capsules, and Terry Naturally-Andrographis EP80 (Extra Strength, 60 Capsules) [29]. There are also tablets manufactured in Thailand with the name of Yanhee Far talai jort tablets or Thailand Fah Talai Jone capsules. On the other hand, in Malaysia there are only three officially registered AP solid dosages, namely HebaBiz *Andrographis paniculata* Plus Tablets (by HAI-O Medicine Sdn Bhd), Nature Spirit *Andrographis paniculata* 100 mg capsule (by Nature Spirit Science Research Sdn Bhd), and ALMAA *Andrographis paniculata* 450 mg Capsule (by VM Almaa Herbal and Health Care Sdn Bhd). Most of these tablets are widely sold as supplements or herbal medicines.

The therapeutic efficacy of “Kan Jang^®^” tablets (manufactured by Swedish Herbal Institute (SHI)) which contains *A. paniculata* extract (APE) was studied in a double-blinded study in 1995 for the treatment of common cold [25,30]. The andrographolide content in the tablet was fixed at 4%. The findings showed that patients treated with Kan Jang tablets had a significant reduction of cold, sinus, rhinitis, and headache on the fourth day, with a daily dose of 1200 mg of APE [25,31]. A similar study was carried out with the same tablet in winter seasons on two groups of healthy students (boys and girls age group of 18.20 ± 0.174 to 18.6 ± 0.20), in which one group of the students were given placebo and the other two Kan Jang tablets (100 mg/tablet of 5.6% andrographolide content) for 5 days a week. The students were still getting colds in the first month of consumption, but a significant difference was observed between the two groups after the third month of the study. Only 30% of students treated with Kan Jang tablets had cold compared to 62% of students treated with the placebo. This concluded that there was a 2.1-times decrease in the common cold, which indirectly proved that andrographolide boosted the immunity of the students [32].

On the other hand, KalmCold™ is an APE capsule manufactured from India and it is used for uncomplicated upper respiratory tract infections. The capsules consisting of 100 mg APE with 31.30% of andrographolide. A clinical study showed that the capsule has the potential to suppress common cold symptoms, such as nasal discharge, expectoration, cough, sore throat, headache, fever, fatigue/malaise, earache, and disturbance during sleep, which were seen to have a downward trend [26,31,33,34]. Besides AP extracts, there are also solid dosage formulations with andrographolide at a dose of 10 mg/kg for HIV positive patients (PN355, Paracelsian, Inc., Ithaca, NY, USA). Phase I trials confirmed that APE extract can cause HIV-induced cell cycle dysregulation inhibition, leading to an increased count of CD4+ lymphocyte from 405 cells/mm^3^ to 501 cells/mm^3^ in HIV-1 patients [35].

In addition, the potential of AP in combination with other antibiotics has been studied over the years for pharyngotonsillitis, uncomplicated acute URTI, sinusitis, common cold, influenza, and HIV [26,36,37]. In these cases, we notice the amount of dose consumed by patients is about 3–4 tablets (85–500 mg) 3–4 times daily, especially if it is in the extract form. Hence, the daily intake is between 255 mg to 2000 mg, which may cause side effects [38].

Even though the European Medicines Agency (EMA) states that AP extracts are safe, with no acute or genotoxicity data, high doses are reported to cause reproductive toxicity [24,39]. Vomiting, diarrhea, urticaria, and epistaxis were observed in a KalmCold treated group, even with a total dose of 200 mg a day [33]. In addition, nausea, constipation, intense headache, dizziness, drowsiness, and chest discomfort have been reported with APE consumption [39]. AP extracts are known to have other bioactive components such as viz., 14-deoxy-11, 12-didehydroandrographolide, which has been reported to lead to dose-dependent hypotension [7,33]. Pharmacovigilance analysis by the Therapeutic Goods Administration (TGA) disclosed that hypersensitivity or allergic reactions are the most common adverse effects found with *A. paniculata* intake [40].

A pharmacokinetic study by Panossian et al., revealed that the bioavailability of andrographolide declined by four times (0.21: 0.91) with a 10-fold dose increment (200 mg/kg: 20 mg/kg). A half-life of 6.67 h at a dose of 20 mg/kg was achieved for oral administration of APE. Once 90% of andrographolide was absorbed into the bloodstream, the remainder started to penetrate into the tissues intensively and became metabolized and bound to blood proteins [41]. Thus, high dosage regimens should be studied thoroughly, as increase of APE dose activates either the rate or extent of andrographolide absorption into the tissues or the metabolism rate in the body [42,43].

Formulations in the form of AP extracts should also be re-evaluated for their andrographolide content for human consumption, for better therapeutic efficacy. Some drawbacks of AP solid dosage forms are their poor oral bioavailability, resulting from poorly water-soluble AP extract or andrographolide [44]. Furthermore, the consumption of high doses of AP extract may lead to patient incompliance and difficulty in swallowing, especially in children and elderly patients [45]. Solid dosage forms will also pass through the hepatic first-pass metabolism and may lead to gastrointestinal discomfort.

### 2.2. Liquid Dosage Forms: Extracts, Syrups, Tinctures, and Injections

Natural products or herbal extracts can also be delivered in liquid dosage form, either through oral or parenteral (injectables) routes [46]. This is a pharmaceutical preparation that contains active ingredients and excipients dissolved or suspended in a compatible solvent. There are three types of liquid dosage systems: single-phase system (monophasic), two-phase system (biphasic), and multi-phase system. Single-phase systems are clear and homogenous solutions containing one or more APIs dissolved in one or more type of solvents forming syrups, elixirs, or mixtures; while two-phase or multiphase systems are of one phase (solid particles, oil droplets, vesicles) dispersed in another phase forming suspensions or emulsions [21,47]. In general, liquid dosage forms are easier to consume for the children and elderly, owing to facile dose adjustments, and have a faster onset of action and absorption rate compared to a solid dosage [48]. However, liquid preparation requires additive ingredients or excipients such as stabilizers, suspending agents, vehicles, preservatives, solubilizers, emulsifying agents, flavors, and colors; in the case of AP, sweetening agents seems to be compulsory to mask its bitter taste [49,50].

Various liquid formulations of AP have been marketed globally. For instance, SBL’s Kalmegh Syrup (500 mL), Homeopathic Mother Tinctures Kalmegh Q (30 mL, 100 mL), Vasuliv syrup, HAPDCO Kalmegh’s Drop, and Hahnemann Laboratory’s Kalmegh Drop (15 mL) are available in India. In China, Beijing Haierfu Pharmaceutical Co., Ltd. produces Fu Fang Shuang Hua liquid dosages for acute tonsillitis oral administration [39]. Besides these, “Yamdepieng” and “Chuanxinlian Ruangas” are also available in liquid dosage form for parenteral administration through injections [28]. On the iHerb and Walmart websites, there are only two types of liquid formulation available: Herb Pharm, Andrographis Tincture, 1 fl oz (30 mL), and Chuan Xin Lian, *Andrographis paniculata* Tincture, (Herbal Terra, Honolulu, HI, USA) 4 oz as supplement. This concentrated liquid formulation is recommended to be consumed by mixing 1 full dropper with 2 oz. of water or juice. In addition, there are AP liquid preparations in the form of injections for animals such as horses, pigs, cattle, sheep, dogs, cats, etc., which are officially approved and GMP certified for veterinary applications. For example, South Ranch or Nanhua Qianmu *Andrographis paniculata* liquid injection and Chuanxinlian injections for animals are sold through online platforms such as Shopee and Lazada. *A. paniculata* extract or andrographolide powders or granules also available on the market, and these need to be mixed or dissolved in water prior to consumption. Studies reported that the pharmacokinetics and pharmacodynamic activity remains the same as of the tablets or granules dispersed in water, but with better absorption and a faster onset of action, due to the absence of excipients, unlike in solid dosage forms [51].

A group of researchers from the Bangladesh Council of Scientific and Industrial Research (BCSIR) showed the production process of AP syrups. Unlike the solid dosage form, the dried extracted powders or granules of *A. paniculata* were mixed with sugar solution, sodium metabisulphite, and citric acid and subjected to 600–700 °C, in order to obtain the syrups. The pharmacological activity and toxicity of the prepared liquid formulation was tested using a mouse model. No obvious or adverse effects were noted for the hematological and biomolecule parameters (RBC, WBC, serum cholesterol, serum protein, serum creatinine, and serum glucose) in the treated mice, as compared to the non-treated group [52]. There are also AP syrups made using honey, known as Oxymel. The addition of sugars or honey to the AP masks its bitterness and enhances patient compliance [53].

It should be emphasized that AP injections are only marketed for animals and not for humans. However, there are a few intravenously delivered AP studies reported by T. Yang and his team that have investigated the pharmacokinetics of andrographolide and its metabolite 14-deoxy-12-hydroxy-andrographolide (DEO-AG) when delivered intravenously (5 mg/kg) in a rat model. DEO-AG was first discovered in rat serum as a phase 1 metabolite after gavage feeding of AG in rats. The pharmacokinetics of AG and DEO-AG, both had a swift decrease in the serum concentration, with subsequent steady decline in the elimination phase. However, it was observed that the andrographolide elimination half-life was 403.19 ± 108.84 min slower than 14-deoxy-12-hydroxy-andrographolide (t½ 205.00 ± 44.93 min). While the mean residence time (440.95 ± 102.73 min) of andrographolide was longer and its high surface distribution volume had an apparent volume of distribution of 53,649.81 ± 16,340.66 mL/kg. Similarly, another study of 20 mg/kg intravenously administered AP extract showed an exponential decline to 0.11 µg/mL after four hours, with the andrographolide concentration at 5.62 µg/mL rat blood plasma at 0.25 h. It had a Vd of 0.27 l, clearance at 0.24 mL/min, half-life of 1.31 h, and AUC 0-∞ of 7.92 µg.h/mL [41]. This shows that andrographolide is accumulated in tissues, or that the activation of its metabolism takes place following intravenous injection, as andrographolide begins to infiltrate intensively into the tissues after maximum absorption and will be metabolized and bound to blood proteins [41,54].

Considering the toxicity of AP, a WHO monograph had presaged the use of crude extract injections, due to potential anaphylactic reactions [40]. However, liquid dosages have a rapid absorption and achieve a more rapid therapeutic concentration than solid dosages when delivered orally. Liquid dosages also have an advantage in delivering medications to children and are suitable for i.v and i.p routes for unconscious patients [55]. Additionally, fluids reach systemic circulation easier and more quickly, as dissolution is not needed before absorption [56].

### 2.3. Semi-Solid Dosage Forms: Creams, Pastes, Ointments, Gels, Poultices, and Foams

Pharmaceutical semi-solid preparations are topical dosage forms that offer localized and systemic therapeutic effects at the site of application, i.e., skin, accessible mucous membranes (nasal cavity or vagina), cornea, buccal tissue, rectal, or any outer parts of the body. These dosage forms are non-dehydrating, non-hygroscopic, non-greasy, and non-gritty, with a smooth and stable texture [57]. Semi-solid formulations are commonly used for cosmetic purposes, as a protective layer or even for therapeutic needs, to treat certain pathological conditions. Widely available semi-solid preparations can be categorized into creams, pastes, ointments, gels, plasters, poultices, and foams [58]. This is a three-phase system in which the API is dissolved in one or both phases (water and oil) [57]. Hence, delivering AP topically using semi-solid dosage forms can benefit from lower side effects or toxicity levels, due to its local or site-specific actions on the affected area, on top of avoiding the first-pass and hepatic metabolism [59,60]. Semi-solid formulations are highly recommended for bitter drugs and have a better stability compared to liquid dosage forms. Examples of currently existing semi-solid dosage forms of AP are Fiog Kang Wang Ke Ji *Andrographis paniculata* Cream 10 g (skincare set) and Bianca Rosa Andrographis 3% Cream (2 oz) 50 mL.

In a study conducted by Suwalee Thawornrungroj and his team, AP was formulated into a gel formulation and given as an antimicrobial adjunct for periodontal pocket localization. The clinical and antimicrobial effectiveness of AP gel was investigated after scaling and root planning (SRP). The SRP and AP gel treated group showed the highest improvement in the depth of probing pocket (2.78 + 0.71 mm), level of clinical attachment (3.22 + 0.97 mm), gingival index (1.00 + 0.25), and the bleeding on probing percentages (46.88 %), as compared to the SRP gel base and SRP only treated groups. At baseline, *P. gingivalis* was not detected in five subjects after 3 months, out of eight subjects noted at baseline, proving its strong antimicrobial activity for the treatment of chronic periodontitis [61]. AP gel and AP extract has also been reported to promote human periodontal ligament (PDL) cell differentiation into bone-forming cells with the activity of alkaline phosphatase and formation of mineralized nodule in gingival tissues at the localized AP gel site. AP gel is also known to treat acne and it has the best antimicrobial effect at 2.5% (*w*/*w*) against *S. aureus* and *C. albicans*, with 5.0% (*w*/*w*) against *P. acnes* and *S. epidermidis* [62,63].

Other than gel, AP ointments are also present and were tested by the Ministry of Health in Indonesia, formulated using an ethanolic extract of *A. paniculata*. The experiment that aimed to investigate the antibacterial properties and physical nature of the ointments resulted in a blackish green semi-solid ointment formulation with a pH of 6, which is in line with the skin’s normal pH range (4.5–6.5) [64,65]. Additionally, a homogeneity test confirmed it as a homogenous semi-solid dosage with high adhesion and dispersive power. An adhesion test (82 s) revealed that the ointment would attach to the skin longer, providing a better effect; meanwhile, the dispersive power (5.6 cm) increased with a wider contact area of the drugs with the skin, for a higher drug diffusion [66]. AP has also been formulated as a cream. Methanolic and aqueous extract of AP cream were formulated into a cream, using shea butter via an oil-in-water emulsion. The antimicrobial properties of both aqueous and methanolic extract of AP and the stability of the creams in different storage conditions were evaluated. No significant differences in both extracts against the test organisms were found (*Escherichia coli*, *Pseudomonas aureginosa*, *Staphylococcus aureus*, *Candida albicans* and *Aspergillus niger*), except for the methanolic extract cream’s susceptibility against *Aspergillus niger*, which showed a higher antimicrobial activity. All the prepared APE formulations were within a pH range of 6–7. Shea butter was also discovered to have a synergetic effect in combination with AP against microbes and microorganisms [67].

Even though AP semi-solid dosage forms have no reported toxicity or adverse effects, the semi-solid formulations can cause allergies or skin irritation, depending on the patient’s topical condition [68]. Another major drawback of these dosage forms is the inaccuracy of the dose delivered and being physicochemically less stable compared to solid dosage forms [69].

### 2.4. Gaseous Dosage Forms: Aerosols, Sprays, and Inhalers

In 1956, the first pressurized metered dose inhaler (MDI) was introduced by Riker Laboratories, Inc., and this marked the beginning of the modern pharmaceutical gaseous dosage form [70]. Types of gaseous dosage forms include aerosols, sprays, vaporizers, inhalers, aero dispersions, nebulizers, and atomizers [71,72]. To date, there have been minimal resources or publications on the gaseous dosage forms of AP. There are also no products officially approved by the FDA with a gaseous dosage form.

Researchers have investigated the anti-asthmatic properties of aerosolized andrographolide. Asthma development is associated with the activation of nuclear factor (NF)-kB, while andrographolide was shown to hinder NF-kB activity, both in humans and rats. Aerosol administration of andrographolide (0.1, 0.5, and 1 mg/kg) acted in a dose-dependent manner and resulted in decline of total cell and eosinophil counts in the BAL fluid, when tested using an asthma mice model. A significant reduction in lymphocyte, neutrophil, and macrophage counts was observed with mice treated with a high dose of andrographolide (1 mg/kg). Inhalation of andrographolide leads to attenuation of eosinophilia and airway mucus formation in OVA-induced lung tissues; reduced mRNA expression of E-selectin, Muc5ac chitinases, and inducible nitric oxide synthase; as well as airway hyperresponsiveness to methacholine. In nuclear extracts from OVA-challenged mice lung tissues, andrographolide inhibited p65 nuclear translocation and DNA-binding activity [73]. Thus, these findings show that andrographolide inhaled through an aerosol has a high therapeutic value in asthma treatment.

The limited AG gaseous products available show that there is a gap in research related to gas dosages. Gaseous dosage forms could lead to a better efficacy, with a rapid onset of action with a limited dose, and with localized or targeted delivery, which results in high drug concentration to the target organ, while lowering the existing dose using conventional routes [74,75]. Nevertheless, this could also limit the dose delivered, due to airway symptoms and cause irritant effects to airways, besides being costly [76].

## 3. Advances in *A. paniculata* Nano-Formulations

**Figure 4:**
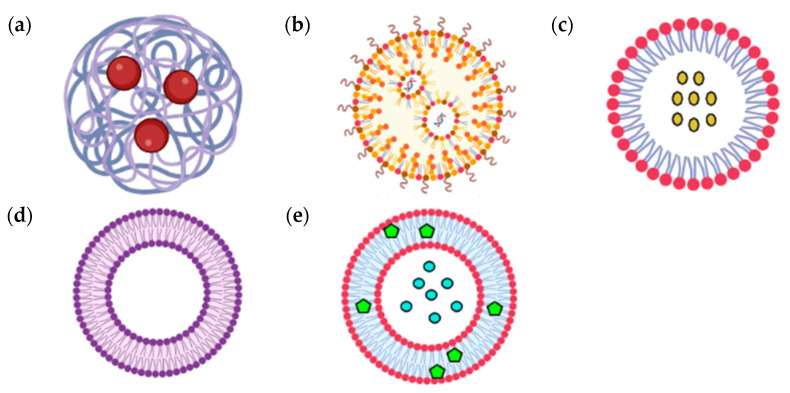
Schematic representation of various nano-particulate systems investigated with *A. paniculata* and andrographolide. (**a**) Polymeric nanoparticles, (**b**) solid lipid nanoparticles, (**c**) polymeric micelles, (**d**) liposomes, and (**e**) niosomes (created using BioRender.com, accessed on 1 September 2022).

As discussed above, AP formulations still have a low bioavailability, despite their various conventional dosage forms. The commercially available andrographolide or extracts of AP include tablets, capsules, syrups, mixtures, and suspensions, and these products release their API over a short period of time after consumption. Any API or extracts made using these conventional dosage forms dissolve immediately upon administration in the target area and are eliminated quickly. In addition, a rapid increase of API concentration in plasma may lead to nausea, stomach distress, vomiting, and other side effects [77]. In order to maintain effective concentrations of the active components in the plasma, multiple dosing is required, leading to patient incompliance and increased toxicity. Thus, recent advances in herbal formulations coupled with nanotechnology delivery systems have garnered significant attention. Polymeric nanoparticles, lipid nanoparticles (solid-lipid nanoparticles (SLN), nanostructured lipid carriers (NLC), polymer–lipid hybrid nanoparticles (PLN), and gelatin nanostructured lipid carriers (GNLs)), liposomes, niosomes, micelles, hydrogels, and nano-emulsions are some of the well-studied nanotechnology platforms, which have been investigated for the delivery of andrographolide and extracts of AP [78,79]. Table 1 summarizes the novel AP extracts and AG delivery through different types of nanoparticulate systems (Figure 4), as well as the key findings over the years 2017–2022.

Herb-based nano-formulations have enhanced drug targeting, in which they translocate the drugs in the required quantity to the diseased cells or tissues in the body [80]. For instance, polymeric nanoparticles (Figure 4a) can be produced with polymers (natural or synthetic) such as PLGA, PLA, chitosan, and Eudragit S 100 as the carrier, to entrap the poorly soluble AG for better distribution in the targeted area. Polymeric nanoparticles can be classified into two types, which are nanospheres (drug distributed homogenously in a matrix system) and nano-capsules (drugs positioned in the core of a polymer shell). In any polymeric formulation of nanoparticles, the type of polymers and solvent used to dissolve the drug is an important factor. An anticancer study conducted by Oseni et al. investigated different PLGA ratios and organic solvent factors (chloroform, ethyl acetate, acetone, dichloromethane) for formulating AG nanoparticles. Andrographolide was identified as highly soluble in acetone, followed by ethyl acetate, and had a high level of encapsulation in 50:50 PLGA. The AG loaded PLGA nanoparticles had a particle size of 135 ± 4 nm, zeta potential of −11.7 ± 2.4, and drug loading of 2.6 ± 0.6% *w*/*w*. The sustained release profile of this formulation confirmed a superior anticancer activity, as compared to the free drug, for 48 h in a metastatic cell line of breast cancer. Hence, this is promising, in that polymeric nanoparticles could improve the bioavailability and delivery of AG compared to the conventional dosage form [81].

Besides polymeric nanoparticles, biosynthesized or biogenic metallic nanoparticles such as silver (Ag) or gold (Au) nanoparticles are produced using green formulations and a cost-effective method. AP extracts formulated into green silver nanoparticles go through bioreduction, forming a dark brown colored solution. Surface morphology studies using SEM and TEM showed a uniform spherical shape with an average size of 11 nm. An antifilarial study using AP-AGNPs on adult female filarial parasites reported a LC50 of 11.6 μg/mL and also showed a significant reduction in parasite levels. These novel green nanoparticles also induced oxidative stress with mitochondrial-mediated apoptosis for a superior antifilarial efficacy, compared to the free AP extract treated group, when tested in adult filarial parasitic worms [82].

Liposomes (Figure 4d) are small spherical shaped lipoid vesicles fabricated from cholesterol and various natural phospholipids (soybean phosphatidylcholine, synthetic dialkyl, trialkyl lipids). The Food and Medication Administration (FDA) has approved several liposomal-based drug delivery systems for medical treatment. An in vivo study showed that rats administered with inhalable AG liposome demonstrated a powerful anti-*S. aureus* effect of pneumonic compared to the AG only group with a ten-times higher dose or with penicillin (antibiotic). Liposome dry powder significantly reduced pro-inflammatory cytokines (TNF-α, IL-1) and restricted IκB-α phosphorylation, in order to modulate the immune response against antibacterial effects and downregulate the inflammatory response [83].

Niosomes (Figure 4e) is a non-ionic surfactant vesicle that can reduce systemic absorption of drugs, for better biocompatibility and therapeutic potential [84]. Niosomes also have the advantage of evading metabolizing enzymes better than other vesicles. Niosomes form closed bilayer structures when non-ionic amphiphiles self-assemble in an aqueous medium. Non-ionic surfactants, such as polyoxyethylene alkyl ethers, polyoxyethylene sorbitan monoesters (Tween 20, 60, 61, and 80), and sorbitan monoesters (Span 20, 40, 60, and 80) have been used to make niosomes. A niosomal gel formulation containing *A. paniculata* ethanol extracts showed a 100% wound recovery, which was due to greater cell viability and wound closure towards fibroblast cells [85].

Micelles (Figure 4c) is also known to be an effective carrier system in drug delivery. Polymeric micelles are formed when amphiphilic block copolymers are introduced into an aqueous media over their critical micelle concentration value. The hydrophobic inner core of the copolymer moves closer to aggregation and distances itself from water molecules above this concentration, forming polymeric micelles. The unique characteristics of micelles, such as nanoscale size, stimuli-responsive cargo release, structural flexibility, charge switching ability, and self-assembly, could overcome the drawbacks of the poor bioavailability of AG [86,87]. Yao and his team formulated AP extract into mPEG-PLA micelle. The AP extract micelle particles were 92.84 ± 5.63 nm in diameter and also had a high entrapment efficiency and loading capacity of 91.00 ± 11.53% and 32.14 ± 3.02% (*w*/*w*), respectively. In vitro cytotoxicity studies revealed that the particles better enhanced the inhibition of mouse breast cancer 4T-1 cells than free AP, providing a promising approach for cancer regimen activity [88].

Besides the various promising approaches and advantages of nanoparticles, nanoparticulate systems still have drawbacks in terms of toxicity and side effects. For instance, the usage of polymers and micelles may lead to toxic effects occurring at the administration site. While liposomes may occasionally experience oxidation and hydrolysis-like reactions that can lead to side effects upon administration [89,90]. The ability of nanoscale materials to penetrate biological barriers (such as the mucosal barrier, blood–brain barrier, placental barrier, and air–blood barrier) and reach cells and tissues that are typically protected from bulk size materials may also cause toxicity. However, the toxicity and side effects of nanoparticulate drug delivery can be controlled with proper dose metrics and a compatible administration route for the chosen drug [91].

Though many studies have discovered that nanoparticulate delivery systems could significantly improve bioavailability and ease the delivery of AG and AP extracts to the targeted area, nanotechnology-based AG and AP extract therapies are still in the experimental stages and the clinical translation of herbal-based nanomedicines is significantly lagging. Hence, further research and global collaboration will lend a helping hand toward the promisingly bright future of AG and AP extract nanocarriers in effective drug delivery systems.

## 4. Conclusions

AP is well known as a natural medicinal plant with wide applications in treating various diseases. However, due to its poor aqueous solubility and the low bioavailability of its active compound, andrographolide (AG), the conventional dosage forms may be unable to deliver the true efficacy of the plant. This biological barrier can be overcome by the incorporation of nanotechnology, which in turn can enhance treatment outcomes. Nanoparticulate delivery systems are advantageous, as they could improve the bioavailability of AG, by improving the water solubility, increasing the residence time of AG in the targeted area, and being used for targeted delivery.

Despite the significant advances in nanotechnology drug delivery platforms, the pharmaceutical industry still lags behind in moving from conventional dosage forms of herbal-based medication to more advanced nanoparticulate drug delivery systems. This is due to a lack of resources in the pharmaceutical industries, especially instruments for scaling-up operations, and the cost involved in nanotechnology product development. There are also major gaps in the pharmaceutical and pharmacokinetic evaluation of advanced nano-medications, which require urgent attention. Perhaps the development of effective plant-based nanomedicines requires a disease-driven approach, in which a solid understanding of the pathophysiology of a disease and the behavior of the nanoparticles in the diseased condition, including their accumulation, distribution, retention, and efficacy, would be thoroughly investigated.

## Figures and Tables

**Table 1:** Types of nanoparticle formulations and their pharmacological and pharmaceutics studies, with their biological activity.

Type of Nanoparticles	Comp	Size and Shape	Formulation Technique	Results	Target Activity/Disease	Ref
PLGA nanoparticle embedded into gelatin-based hydrogel	AG	494.00 ± 4.28 nm (acid end group) and 529.30 ± 7.36 nm (ester end group) (Spherical shape)	Single emulsion solvent evaporation method	The intra-articular drug delivery results in a long-standing sustained release injection (≥2 months) and implantation (≈2 months) for osteoarthritis disease’s local treatment.	Osteoarthritis disease	[92]
PLGA nanoparticles	AG	250 nm (spherical with no visual crevices)	Multiple-emulsion solvent evaporation technique	AG’s bioavailability is enhanced through oral (AUC0-t: 5.37 times and 6.38 times higher in plasma and lung than free-AG) or pulmonary (AUC0-t: 3.2 times and 3.6 times higher in plasma and lung than free-AG) administration. The content of serum IgE, cell numbers, broncho-alveolar lavage fluid levels, NF-κβ suppression demonstrates greatly improved results, with sustained release AG NPs compared to free AG.	Asthma	[93]
Gold nanoparticles	AG	543 nm from plasmon response and 14 nm in TEM (crystalline and spherical)	Facile one-pot technique	The formulation exhibits strong anti-leishmanicidal effects, with macrophage uptake completed within 2 h of introduction, resulting in an IC _50_ value of 19 ± 1.7 µM for wild life, and 41 ± 6 µM for paromomycin or 55 ± 7.3 µM for sodium stibogluconate resistant strains.	Drug resistant VL strains	[94]
Silver Nanoparticles	AP Extract (APE)	53.2 ± 2 nm (Crystalline and spherical)	Priosynthes/green synthesis	APE silver nanoparticles exhibited promising antifungal activity in both *Aspergillus niger* and *Penicillium* sp. (Inhibition diameter: 12 mm and 14 mm)	Antifungal Activity	[95]
Novel Self-Nano Emulsifying Drug Delivery System (SNEDDS)	AG	8.4 ± 0.6 nm to 115.0 ± 2.9 nm (globule)	Shake-flask method, followed by ternary phase diagrams.	AG SNEDDS had a better efficacy in comparison to plain AG, with a 1.26-fold rise in Cmax, 1.2-fold improvement in AUC, and 1.72-fold decline in Tmax in an animal model (New Zealand white rabbits).	NA	[96]
Liposomes	AG	77.91 nm	Injection method	In vivo studies showed rats sprayed using liposome inhalers demonstrated a powerful anti-S. aureus effect of pneumonic instead of AG with 10x higher dose or of penicillin. It reduced pro-inflammatory cytokines (TNF-α, IL-1) and restricted IκB-α phosphorylation, to modulate the immune response against antibacterial effect and downregulate the inflammatory response.	Bacterial pneumonia/Antibacterial and anti-inflammatory activity	[83]
Zinc oxide nanoparticles (nanocrystal)	AP Extract (APE)	96 to 115 nm (spherical, oval, hexagonal)	Biosynthesis/green synthesis method	AP green ZnO nanoparticles have excellent potentials vs. AP leaf extract alone in antidiabetic (IC50: 121.42 lg/mL vs. 149.65 lg/mL), anti-inflammatory (IC50: 66.78 lg/mL vs. 75.42 lg/mL) and antioxidant (62%) activity.	Antidiabetic, anti-inflammatory, antioxidant activity	[97]
Regenerated silk fibroin (RSF) nanoparticles	AP Extract (APE)	200 to 1000 nm (spherical structure)	Green and mild method with additions of ethanol, mPEG-NH2, and freezing RSF-ethanol solution.	For an in vitro drug release profile, 90.9% of APE was released from APE-loaded RSFNPs up to 72 h against APE suspension and APE-saturated solution, which showed a two-fold lower drug release profile. No cytotoxicity was observed and it can attach easily to MDA-MB-231 and Hela cells, demonstrating its anti-proliferative activity.	Lymphatic chemotherapy/anti-proliferative activity.	[98]
NiO nanoparticles	AP Extract (APE)	24 nm (cubic structure)	Microwave-assisted biogenic	Green NiO nanoparticles were evaluated for photodegradation, which resulted in a 88.13% degradation efficiency. It expressed the the lowest IC50 value in an MCF-7 cell line study compared with other metal oxide nanoparticles.	Breast Cancer/anticancer activity.	[99]
Silver nanoparticles	AP Aqueous Extract	123.1 nm	Biosynthesis method	HeLa cell line were used to test the impact of cytotoxicity, which is known to be dose-dependent, with 7.285 μg/mL as the half maximal inhibitory concentration. A total of 24 male mice were injected intraperitoneally with AgNPs (350 g/kg BW) and plant extract (80 mg/kg BW) for 10 days, following tumor formation with a calculated dose (40 µL). The biochemical and hematological indicators studied were restored to near-normal levels in all of the therapy groups, showing AgNPs’ effectiveness against carcinoma cells.	Antitumor activity.	[100]
Silver nanoparticles	AP Methanol Extract	18–70 nm (spherical)	Biosynthesis method	The anticancer efficacy was investigated against neuroblastoma cells and normal Vero cells, which resulted in IC 50 values of 32 and 60 g/mL, respectively. No cytotoxicity (CC _50_ value of 329.29 and 368 µg/mL) was induced by nanoparticles to Vero cell lines.	Anticancer activity.	[101]
Nano-phytovesicular system	Semi-purified AG	395.5 ± 5.80 nm (spherical, unilamellar vesicles with globular shapes)	Liquid dispersion technique with slight modifications	The hyperglycemic condition of rats was significantly protected by the nano-phyto vesicles of semi-purified AP extract, corresponding to 25 mg/kg AG. Compared to free AG (50 mg/kg), the AG vesicles produced superior results in body weight development, oral glucose tolerance test, as well as blood glucose level. Therefore, increased oral absorption, bioavailability, and improved antihyperglycemic action were shown in in vitro and in vivo studies.	Hypoglycaemic activity	[102]
Human serum albumin-based nanoparticles (HSA NPs) and poly ethylcyanoacrylate nanoparticles (PECA NPs)	AG	151.7 to 335.1 nm (spherical)	Thermal and chemical cross-linking method for HSAT AG NPs and HSAC AG NPs and Emulsion polymerization method to yield PECA AG NPs.	An in vitro BBB model based on the hCMEC/D3 cell line was used to examine the potential of free AG and AG-loaded in HSAT and PECA NPs to cross the blood–brain barrier (BBB). In silico research anticipated that free AG would not permeate the BBB model. HSAT NPs enhanced the AG penetration by two-fold, while retaining cell layer integrity, whereas PECA NPs momentarily damaged the BBB integrity.	Inflammation-related pathologies/neurodegenerative disorders	[103]
Niosome	AP Extract (water and ethanol)	125–226 nm (spherical)	Proniosome-derived niosomal dispersion	Ethanol AP extract niosomal gel had a 100% wound recovery rate while protecting tissue from oxidative stress. Towards the end of 14 days, prominent collagen fibers and the development of hair follicles were detected in fibroblasts cells.	Wound healing/antioxidant activity.	[85]
Nanosuspensions	19-tert-butyldiphenylsilyl-8,17-epoxy AG (3A.1) (AG analogue)	less than 300 nm (spherical)	Single step nanoprecipitation (bottom-up) technique	The anticancer activity of NSC derivatives stabilized 3A.1 nanosuspension against colorectal cancer (HCT116) cells was substantially enhanced regarding in vitro cytotoxicity.	Colorectal cancer/anticancer activity.	[104]
Eudragit (R) EPO Based Nanoparticle Suspension	AG	255.9 nm	Nanoprecipitation and lyophilization technique	Compared to pure AG, there was a substantial increase in drug dissolution, with total drug release within 10 min in NPs suspension and re-dispersed in NPs suspension. However, lyophilization slowed the release of AG. In contrast to AG, the NP suspension and re-dispersed NPs suspension showed better hepatoprotectivity for CCl4-induced hepatotoxicity in rats. Histopathological research on liver lesions confirmed the findings.	Hepatic lesions/Hepatoprotective activity.	[105]
Solid Lipid Nanoparticles (SLN)	AG	262 to 278 nm (Spherical)	Emulsion/evaporation/solidifying technique	In vitro findings using hCMEC/D3 cells, an in vitro BBB model, and a permeation test (PAMPA) showed that AG-SLN increased AG permeability and sustainability compared to free AG. Fluorescent SLN were found in brain parenchyma after intravenous delivery in vivo, indicating their ability to transcend the BBB.	Oxidative stress mediated neurotoxicity, inflammation-mediated neurodegeneration, and cerebral ischemia	[106]
Casein Micelle	AP Extract (APE)	-	-	Casein breakdown tends to produce a burst release profile in less than 30 min of simulated gastric fluid digestion (500 of substrate ratio: 1 enzyme optimal (*w*/*w*)). In digestion of simulated gastric fluid, the incorporation of APE in casein micelles delays casein breakdown, leading to a sustained release profile of casein degradation in around 4 h of digestion.	Antidiabetic Activity	[107]
Chitosan Nanoparticles	AG	500 nm to 3000 nm (crystalline)	Ionic gelation technique (spray drying)	The amount of AG dissolved from chitosan nanoparticles during in vitro drug release was enhanced by 6.5-fold compared to AG alone, meanwhile the in vivo antimalarial activity was 1.65-times higher in Plasmodium berghei infected mice.	Antimalarial activity	[108]
Nanoemulsion-based Hydrogel	AG	56.5 ± 1.92 nm (droplet)	Ultrasonication Method	An optimum nanoemulsion of 1.35% of triethanolamine, 9% of propylene glycol, and 34.65% of carbopol was incorporated into a hydrogel base (1:1). A pH of 6.50 ± 0.02 and viscosity cP of 2492.33 ± 36.91 showed the optimum results of the AG-nano-emulsion-based hydrogel.	NA	[109]
Eudragit S 100 nanoparticles	AP Methanolic Extract	300–400 nm (smooth and spherical shape)	Solvent displacement method	It was noted to have an encapsulation efficiency > 75%, as well as an in vitro drug release of >55%, after 8 h.	NA	[110]

## Data Availability

Not applicable.
